# Comparative Analysis of Tyrosine Hydroxylase Amacrine Cells in the Mammalian Retina: Distribution and Quantification in Mouse, Rat, Ground Squirrel and Macaque Retinas

**DOI:** 10.3390/ijms26146972

**Published:** 2025-07-20

**Authors:** Kiyoharu J. Miyagishima, Xiaomin Lai, Amurta Nath, William N. Grimes, Xiyuan Ping, Jeffrey S. Diamond, Morven A. Cameron, Wei Li, Francisco M. Nadal-Nicolás

**Affiliations:** 1 Retinal Neurophysiology Section, National Eye Institute, National Institutes of Health, Bethesda, MD 20892, USA; kiyoharu.miyagishima@nih.gov (K.J.M.); xiyuanping@zju.edu.cn (X.P.); 2 Somatosensation and Pain Unit, National Institute of Dental and Craniofacial Research, National Institutes of Health, Bethesda, MD 20892, USA; xiaomin.lai@nih.gov; 3 Synaptic Physiology Section, National Institute of Neurological Disease and Stroke, National Institutes of Health, Bethesda, MD 20892, USA; amurta.nath@nih.gov (A.N.); william.grimes@nih.gov (W.N.G.); diamondj@ninds.nih.gov (J.S.D.); 4 Eye Center, The Second Affiliated Hospital, School of Medicine, Zhejiang University, Hangzhou 310009, China; 5 School of Medicine, Western Sydney University, Sydney, NSW 2560, Australia; m.cameron@westernsydney.edu.au; 6 Department of Ophthalmology, Faculty of Medicine, University of Murcia and Biomedical Research Institute of Murcia (IMIB-Pascual Parrilla), 30120 Murcia, Spain

**Keywords:** dopamine, tyrosine hydroxylase, amacrine cell, retinal ganglion cells, mammalian retina, monkey, spatial distribution, topography, cell density

## Abstract

Dopaminergic amacrine cells (DACs) are a subclass of amacrine cells that modulate retinal processing and light adaptation by releasing dopamine. Although the role of dopamine is largely conserved, their retinal distribution across mammals remains incompletely characterized. In mice, rats, thirteen-lined ground squirrels (TLGSs), and macaques, we systematically compared the localization, number, and topography of DACs by their expression of tyrosine hydroxylase (TH), a crucial enzyme in the biosynthesis of dopamine. In all species examined, TH+ cells were primarily located in the inner nuclear layer; however, there was a species-dependent influence on their number and distribution. Mice exhibited the highest density of TH+cells but completely lacked displaced TH+cells (dTH+cells) in the ganglion cell layer. Despite interspecies variation in the total number of TH+cells in the retina, the overall density in rats, TLGSs, and macaques was similar. Most species displayed a higher density of DACs toward central retinal regions. However, rats exhibited a distinctive dorsal concentration, particularly among dTH+cells. Although most species examined exhibited a similar ratio of TH+cells to Brn3a+ retinal ganglion cells, TLGSs showed a marked reduction, indicating a potentially diminished dopaminergic modulatory role. Species-specific DAC topographies aligned with specialized visual regions, such as the visual streak in TLGS and the macula in macaques. These results reveal both conserved and divergent features of retinal dopamine circuitry, reflecting evolutionary adaptations to visual processing demands.

## 1. Introduction

The mammalian retina is a highly organized, light-sensitive neural tissue with a laminar structure at the back of the eye that performs the initial stages of visual processing before transmitting this information to the brain. The retina is composed of several neurons—including rod and cone photoreceptors, bipolar cells, horizontal cells, amacrine cells, and retinal ganglion cells (RGCs), that form a complex network that interacts to refine and relay visual signals. Among these cell types are dopaminergic amacrine cells (DACs) which synthesize and release dopamine in the retina, an important neurotransmitter that regulates a broad range of visual functions including visual adaptation to changes in illumination, contrast sensitivity, color perception, circadian rhythms, and even retinal development and vascular function [1,2,3,4,5,6].

DAC synthesis of dopamine is tightly linked to ambient light conditions: dopamine release increases in bright (photopic) conditions and decreases in darkness (scotopic conditions), positioning DACs as key regulators of retinal plasticity [7,8]. A major function of dopamine in this context is to facilitate light adaptation by modulating retinal circuitry in response to changes in illumination. In scotopic conditions, gap junctions facilitate the pooling rod signals to optimize the signal-to-noise ratio (SNR) improving detection of sparse photons. In contrast, under bright light, dopamine acts on receptors expressed by various retinal cells (including horizontal, bipolar, and AII amacrine cells) leading to the uncoupling of gap junctions, and a shift from rod-driven to cone-driven vision [9,10]. This transition enhances visual acuity and contrast, refining both the spatial and temporal resolution of vision under photopic conditions while suppressing rod input [11].

Beyond visual adaptation, DACs play a central role in regulating the retinal circadian rhythm [12]. Dopamine release follows a diurnal pattern, peaking during the day influencing photoreceptor sensitivity and altering the expression of key retinal genes involved in metabolic and functional adjustments [13,14]. This rhythmic control on gene expression is maintained through a reciprocal relationship with melatonin: dopamine suppresses melatonin release during the day, while melatonin inhibits dopamine synthesis at night [15,16].

DACs are also involved in regulation of ocular growth, particularly in axial length, and have been implicated in the etiology of myopia. While reduced dopaminergic activity has been linked to excessive axial elongation in humans—a defining feature of myopia [17,18,19]—the role of dopamine is not fully understood. The association between dopamine and myopia is well established across several animal models, including mice, rats, tree shrews, guinea pigs and chickens [17,20,21,22,23,24,25]. However, findings from mouse models remain inconsistent [26], highlighting the need for further investigation of the specific contributions of dopaminergic signaling to ocular growth regulation. Additionally, dysfunction in retinal dopaminergic signaling has been linked to neurodegenerative and metabolic retinal diseases. In Parkinson’s disease (PD), dopamine deficits in the retina may contribute to visual disturbances in contrast, color, and visual acuity [27,28,29], while in diabetic retinopathy, dopaminergic disruption impairs neurotransmission and visual processing [30]. Given their neuromodulatory and neuroprotective roles, DACs represent a promising therapeutic target.

Despite the conserved role of dopamine in retinal physiology, its distribution, dynamics of release, and receptor expression may exhibit species-specific adaptations. Comparative studies offer insight into how dopaminergic mechanisms support vision across ecological contexts that govern rod or cone dominance. For instance, nocturnal species may show distinct dopaminergic profiles compared to diurnal species, reflecting adaptations in retinal organization and light exposure [31,32]. Such interspecies differences may also reveal mechanisms underlying retinal resilience to neurodegeneration and inform translational strategies for vision preservation [33].

To investigate these evolutionary differences, we analyzed the number and distribution of DACs in the retina by labeling cells expressing tyrosine hydroxylase (TH), the rate-limiting enzyme in dopamine synthesis. TH immunolabeling is commonly used to identify DACs and assessing their density, spatial organization, and morphology [3,34]. In this study, we compared TH^+^cells across four mammalian species—two nocturnal (mouse and rat) and two diurnal (thirteen-lined ground squirrels (TLGSs) and macaques) [35,36]—to assess whether ecological adaptations correspond with dopaminergic cell distribution and structure. Here, we identify conserved and divergent features of TH^+^cells across species, shedding light on the evolutionary significance of dopamine in retinal function and its implications for visual adaptation and disease vulnerability.

## 2. Results

### 2.1. Tyrosine Hydroxylase Antibody Characterization in Dopamine Amacrine Cells

**Figure 1:**
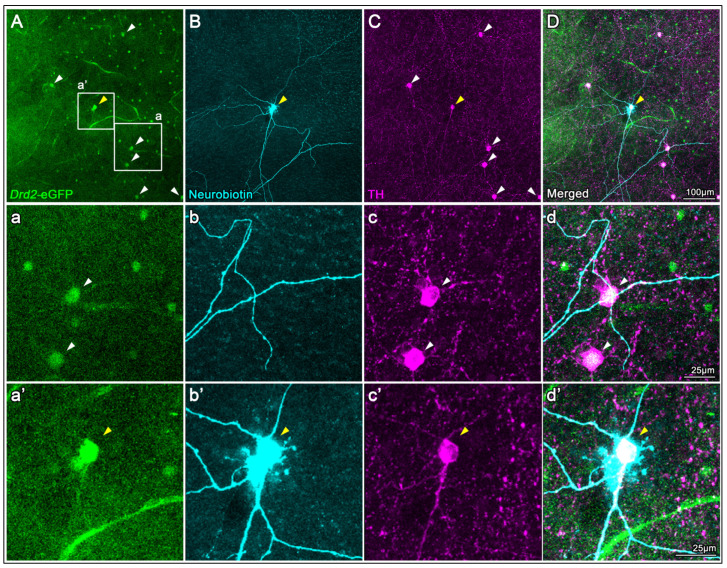
TH antibody characterization in *Drd2*-eGFP mouse retinas. (**A**) Endogenous eGFP fluorescence in a flat-mounted retina from a *Drd2*-eGFP mouse shows two eGFP^+^cell types; DACs are identifiable as large-soma eGFP^+^cells (arrowheads, magnified in (**a**–**d**)). (**B**) A large eGFP^+^cell was intracellularly injected with neurobiotin and subsequently labeled with streptavidin, revealing the characteristic wide-field morphology of a DAC (yellow arrowhead, magnified in (**a’**–**d’**)). (**C**) Immunostaining with a TH antibody selectively labels large-soma cells corresponding to the eGFP^+^DACs (arrowheads in (**A**)), supporting antibody specificity. (**D**) Merged image of all three channels confirms colocalization (magnified in (**d’**)).

To evaluate the specificity of the TH antibody for labeling DACs, we used flat-mounted retinas from *Drd2*-eGFP mice, which endogenously express enhanced green fluorescent protein in 2 types of cells: one with small somas and another with large somas (Figure 1(Aa)) [37]. Among these, DACs can be reliably identified as eGFP-positive cells with large somas (Figure 1(Aa), arrowheads). To confirm their identity, we performed intracellular neurobiotin injections followed by streptavidin immunodetection, which revealed the characteristic wide-field morphology of DACs in one of the large eGFP+cells (Figure 1(Bb’), yellow arrowshead). Immunostaining with the TH antibody selectively labeled a subset of large cells with the typical morphology and distribution of DACs (Figure 1C), supporting the antibody’s specificity for this cell type (Figure 1C,D).

### 2.2. Localization of TH^+^Dopaminergic Amacrine Cells in Retinal Sections from Different Mammalian Species

**Figure 2:**
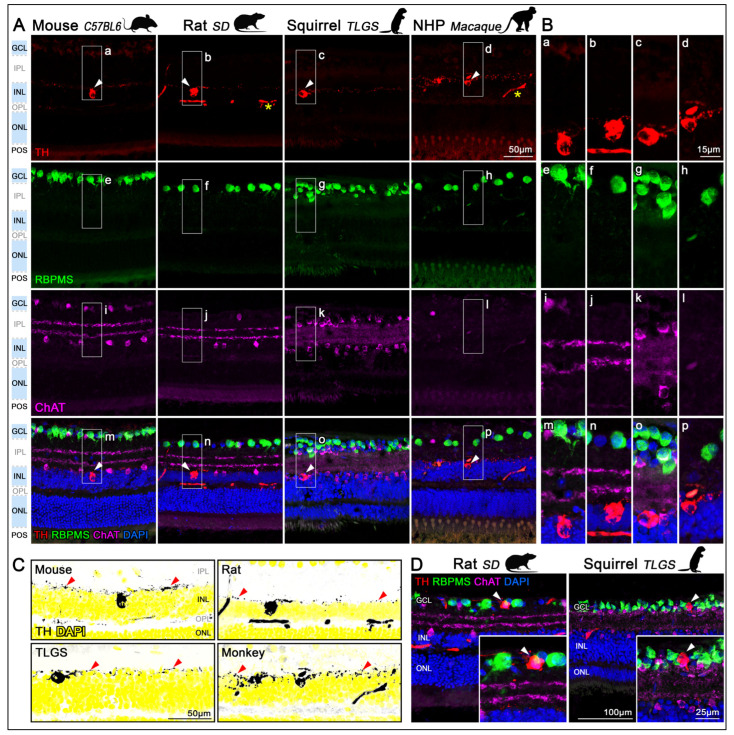
Localization of TH^+^DACs in the retinas of mouse, rat, ground squirrel, and macaque. (**A**) Retinal sagittal sections from mouse (C57BL/6, first column), rat (Sprague-Dawley, second column), ground squirrel (TLGS, third column), and non-human primate (Rhesus macaque, fourth column), immunostained with antibodies targeting distinct neuronal populations and counterstained with DAPI. First row: DACs identified by TH immunolabeling in the inner nuclear layer (INL; arrowheads). Inespecific signals in the vasculature of rat and macaque retinas (asterisk). Second row: RGCs in the GCL labeled with RBPMS. Third row: Starburst amacrine cells labeled with ChAT, located in both the INL and displaced to the GCL. Foruth row: Merged image showing all immunolabels and DAPI (blue) counterstain. TH^+^DACs are preferentially located in the innermost row of INL. Images were acquired from the medial region of the retina (n = 3 retinas per species). (**B**) Detailed magnifications showing TH (**a**–**d**), RBPMS (**e**–**h**), ChAT (**i**–**l**), and the corresponding merged images (**m**–**p**), all of which correspond to the insets (**a**–**p**) in panel (**A**). (**C**) High-contrast grayscale images of TH labeling, corresponding TH^+^cells ((**A**), first row), with DAPI counterstaining in yellow illustrate the laminar distribution of the TH^+^ dendrites within the outer layer of the IPL (red arrowheads). (**D**) Representative cross-sections showing displaced TH^+^cells in the GCL of rat and TLGS retinas (arrowheads). GCL, ganglion cell layer, IPL, inner plexiform layer; INL, inner nuclear layer; OPL, outer plexiform layer; ONL, outer nuclear layer; POS, photoreceptor outer segments. All images across species were acquired using identical magnification ensuring consistent scales.

In retinal cryo-sections, TH labeling was primarily localized to the inner nuclear layer (INL) in mouse, rat, TLGS, and macaque retinas (Figure 2A,B, first row). Across all species examined, TH^+^cells appeared sparsely distributed within the INL and were largely absent from the ganglion cell layer (GCL), identified using RPBMS as a selective marker for RGCs (Figure 2A,B, second row). Co-labeling with choline acetyltransferase (ChAT), a known marker for starburst amacrine cells, delineated the inner and outer sublaminae of the inner plexiform layer (IPL). Notably, TH^+^ dendrites arborized predominantly in the outer sublamina of the IPL, where they extended continuously across the entire retinal surface (Figure 2, third and fourth rows). Enhanced visualizations of TH^+^ dendritic arborizations within the outer layer of the IPL (red arrowheads) are shown by high-contrast grayscale images of TH labeling with DAPI counterstaining pseudocolored in yellow (Figure 2C). An in-depth analysis demonstrated the existence of a small population of displaced TH^+^cells within the GCL. Indeed, RBPMS staining confirmed that dTH^+^cells are not RGCs, as illustrated in representative examples from rat and TLGS retinas (Figure 2D).

### 2.3. Density of TH^+^Dopaminergic Amacrine Cells Across Retinal Regions

**Figure 3:**
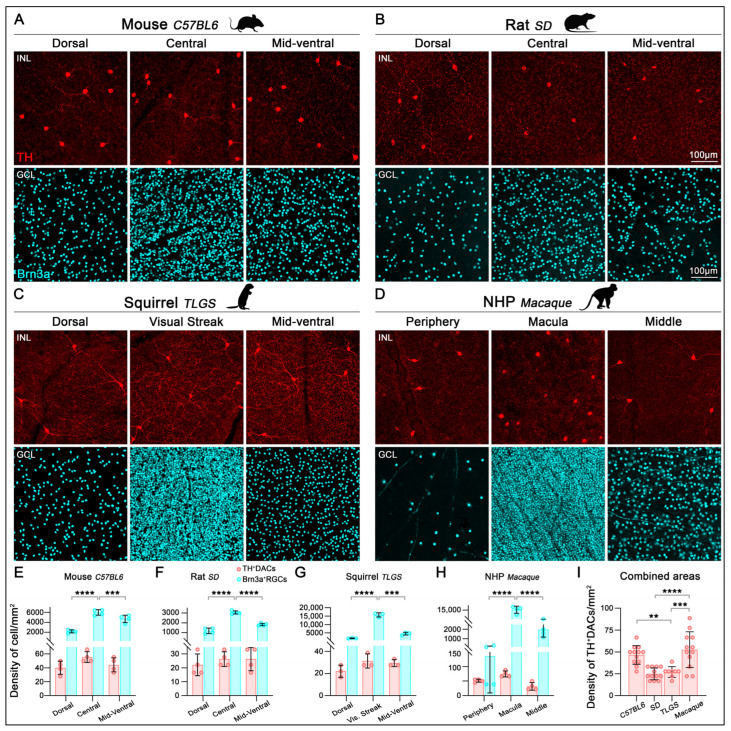
Localization and quantification of TH^+^cells and Brn3a^+^RGCs in the retinas of mouse, rat, ground squirrel, and macaque. Representative images from flat-mounted retinas sampled along the dorso-ventral axis (dorsal/peripheral, central (visual streak or macula), and mid-ventral regions, depending on the species) and immunostained with antibodies against TH and Brn3a to label DACs (INL) and RGCs (GCL), respectively. Images correspond to mouse (C57BL/6, (**A**)), rat (Sprague-Dawley, (**B**)), ground squirrel (TLGS, (**C**)), and non-human primate (Rhesus macaque, (**D**)). TH immunolabeling reveals a relatively uniform distribution of DACs across the retina, in contrast to the regionally variable densities observed for Brn3a^+^RGCs. (**E**–**H**) Bar graphs show a significant increase in Brn3a^+^RGC density (cyan) in the central retina (visual streak or macula) compared to dorsal and ventral regions in mouse (**E**), rat (**F**), TLGS (**G**), and macaque (**H**). In contrast, TH^+^cells (red) show no significant regional variation in density (**E**–**H**). (**I**) Bar graph comparing the average TH^+^cell density (combined from dorsal, central, and ventral regions) across species, with significantly higher densities observed in the mouse and macaque retinas (for macula regions). Data presented as mean ± SD, statistical analysis was performed using one-way ANOVA with Tukey’s post hoc test for multiple comparisons. Statistical significance is indicated as follows: *p* < 0.01 (**), *p <* 0.001 (***), and *p* < 0.0001 (****). Scale bar is consistent across all images.

We next evaluated the density of TH^+^cells in relation to RGCs across various retinal regions (Figure 3A–D). High-resolution imaging along the dorsoventral axis in rodents or periphery-to-center axis in non-human primates (NHPs) revealed significant regional variation in RGC densities across the mouse, rat, TLGS, and macaque retinas (Table 1; Figure 3E–H, cyan bars). In contrast, the density of TH^+^cells remained uniformly low across regions, with only a slight, nonsignificant increase toward the central retina (Table 1; Figure 3E–H, red bars). Overall, the TH^+^cell-to-RGC ratio after examining three different retinal regions (dorsal/periphery, center/macula, midventral) was approximately 1:91, 1:81, 1:265, and 1:112 in mouse, rat, TLGS, and macaque retinas, respectively (Table 1). To compare DAC distribution across species, we calculated the average TH^+^cell density across all three sampled retinal regions (dorsal/periphery, center/macula, and mid-ventral) for each species (Figure 3I). This analysis revealed that macaques and mice exhibit the highest average TH^+^cell densities, while rats show the lowest; however, this difference was not statistically significant when compared to the TLGS.

**Figure 4:**
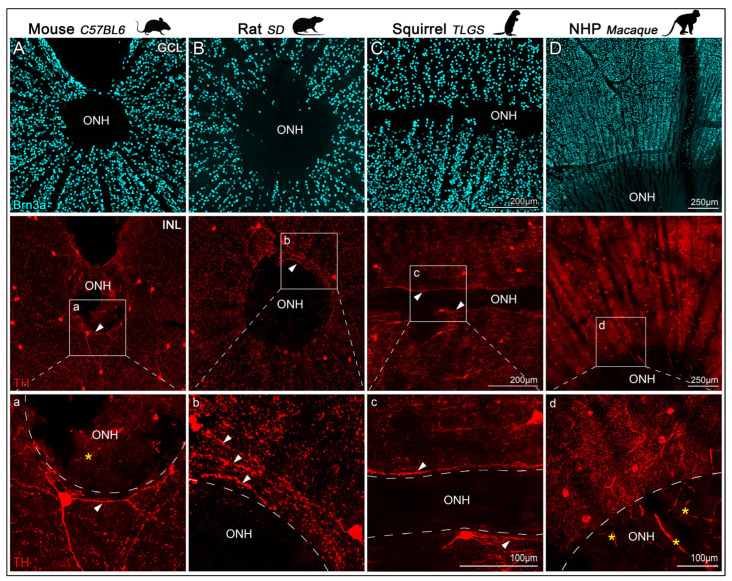
Confocal images of TH^+^cells and Brn3a^+^RGCs near the ONH in retinas from mouse, rat, ground squirrel (TLGS), and macaque**.** Representative images of flat-mounted retinas, focused on the optic nerve head (ONH), showing immunolabeling of Brn3a and tyrosine hydroxylase (TH) to visualize RGCs in the GCL (top row) and DACs in the INL (second row) from: mouse (C57BL/6, (**A**)), rat (Sprague-Dawley, (**B**)), ground squirrel (TLGS, (**C**)), and non-human primate (Rhesus macaque, (**D**)). Panels (**a**–**d**) show higher magnification views of the TH^+^ dendritic arbors surrounding the ONH, taken from the regions highlighted in the second row. TH^+^ dendrites form a dense plexus encircling the ONH but do not extend into it (arrowheads). Image dimension, resolution and scale bars are comparable across images for mouse, rat, and squirrel. Note that auto-fluorescent blood vessels within the ONH appear in some images but do not represent true TH signal (asterisk).

TH^+^ dendrites were also observed surrounding the optic nerve head (ONH, Figure 4, top row). Although TH^+^ somas were sparse near the ONH, their dendritic processes covered the surrounding retinal surface and extended from the far periphery (images not included), closely encircling the axon bundles exiting the retina (Figure 4, second and third rows). In this region, TH^+^ dendrites formed a circular network that appeared more elaborate in rat and macaque than in mouse. The ONH region, characterized by the absence of RGC somas, was also deprived of TH^+^ dendrites. Interestingly, despite the distinctive horizontal elongation of the ONH in TLGS, TH^+^ dendrites displayed a comparable peripapillary distribution (detailed in Figure 4, third row).

### 2.4. Total Number and Density of TH^+^Dopaminergic Amacrine Cells

**Figure 5:**
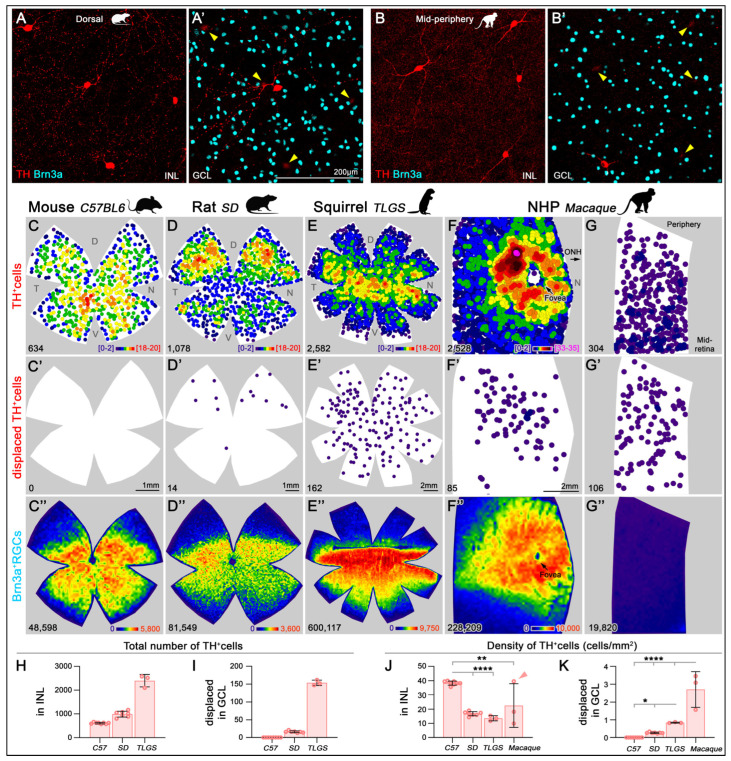
Topographical distribution of TH^+^cells and Brn3a^+^RGCs in mouse, rat, ground squirrel (TLGS), and macaque retinas. Representative image of TH^+^cells in the INL of the dorsal rat retina (**A**) and the mid-periphery of the macaque retina (**B**). Corresponding frames focused on the GCL showing a displaced TH^+^cell and Brn3a^+^RGCs for rat and macaque retinas ((**A′**,**B’**), respectively). Arrowheads point to the faint labeling of the TH^+^ somas from the INL visible in panel (**A**,**B**), respectively. (**C**–**G**) K-nearest neighbor maps showing the spatial distribution of TH^+^DACs in the INL for mouse, rat, TLGS, and macaque retinas (central and mid-peripheral retina). (**C′**–**G′**) Corresponding K-nearest neighbor maps of TH^+^dDACs located in the GCL. Color scales (bottom right of top panels) indicate local density ranging from [0–2 neighbors/cell] (purple) to [18–20 neighbors/cell] (red) or [33,34,35] (pink) within a 525 µm radius in mouse, 700 µm in rat and TLGS, or within a 415 µm radius in Macaque, respectively. (**C**″–**G**″) Isodensity maps showing the topographic distribution of Brn3a^+^RGCs in the same areas. Color scales range from 0 [purple] to 5800 RGCs/mm^2^ in mouse, 3600 in rat, 9750 in TLGS, or 10,000 in macaque [red]. The bottom left of each map indicates the total number of TH^+^cells or Brn3a^+^RGCs per retina. (**H**,**I**) Bar graphs showing significantly higher total numbers of TH^+^cells (**H**) and dTH^+^cells (**I**) in TLGS compared to rat, and in rat compared to mouse. (**J**) Bar graph showing significantly higher average TH^+^cell density in mouse retinas compared to rat and TLGS. Macaque retinas exhibit high regional variability; the sample marked with a red arrowhead includes the macula, fovea, and surrounding areas—its average density is lower than that of the macula alone (evident in the K-nearest neighbor in panel (**F**)). (**K**) Bar plot showing a progressive increase in the density of dTH^+^cells with increasing species complexity. Data are presented as mean ± SD, with statistical significance assessed using one-way ANOVA with Tukey’s post hoc test for multiple comparisons. Statistical significance is indicated as follows: *p* < 0.05 (*), *p* < 0.01 (**), and *p* < 0.0001 (****). D, dorsal; T, temporal; N, nasal; V, ventral.

To quantify the total number and spatial distribution of TH^+^cells and Brn3a^+^ RGCs across the entire retinas of mouse, rat, and TLGS, as well as partial macaque retinas, we used wholemount immunolabeling (Figure 5A,A′) and an automated cell-counting algorithm [38] (Figure 5B). Manual counts of TH^+^cells in mouse, rat, and TLGS retinas yielded averages of 616 ± 33, 993 ± 123, and 2400 ± 262, respectively (Table 2; Figure 5C–F,H). When corrected for retinal area, the average density of TH^+^cells was 38 ± 1, 17 ± 2, and 14 ± 2 cells/mm^2^ in mouse, rat, and TLGS retinas, respectively, while macaque retinas showed an average density of 29 ± 18 cells/mm^2^ (Table 2; Figure 5J).

Within the GCL, displaced TH^+^cells (dTH^+^cells, Figure 5, second row) were infrequent compared to orthotopic TH^+^cells in the INL. Whole retina quantifications showed 0, 16.1 ± 2.5, and 153.7 ± 7.6 dTH^+^cells in mouse, rat, and TLGS retinas, respectively, making up 0%, 1.6%, and 6% of the total DACs (Table 2; Figure 5I). In macaque retinas, dTH^+^cells constituted roughly ~12% of the total quantified TH^+^cells (Table 2).

In whole retinas, the average number of Brn3a^+^RGCs (Figure 5, third row) was 46,497 ± 1768 (mouse), 84,478 ± 3268 (rat), and 607,661 ± 17,557 (TLGS), with corresponding densities of 3084 ± 117, 1652 ± 106, and 3754 ± 171 RGCs/mm^2^, respectively. In macaque samples, the RGC density averaged 1708 ± 2345 cells/mm^2^ (Table 2). The TH^+^cell/RGC ratio across whole retinas was consistent across species, except in TLGS, which showed a lower ratio of approximately 1:76, 1:85, 1:253, and 1:79 in mouse, rat, TLGS, and macaque (partial) retinas, respectively (Table 2). These values are more representative than those calculated by area (Table 1) but they are largely similar, except for macaque.

### 2.5. Topography of TH^+^Cells

k-neighbor mapping of TH^+^cells across entire retinas (Figure 5, first row) confirmed their low density in all species compared to RGCs (Figure 5, third row). Mice exhibited the highest average DAC density, followed by macaque (notably in macular regions), while rat, TLGS, and non-macular macaque retina showed similar values (Figure 5J). dTH^+^cells in the GCL displayed greater interspecies variability, ranging from virtually absent in mouse to relatively enriched in rat, TLGS, and particularly in macaque (Figure 5K).

The topographic distribution patterns of orthotopic TH^+^cells varied across species. In mice, TH^+^cell density was highest in the central retina, especially in the mid-ventral region, with lower density in the dorsal retina (Figure 5C). In rats, TH^+^cell density increased in the middle dorsal and ventral retinas, peaking in the mid-dorsal retina (Figure 5D). TLGS retinas showed the highest TH^+^cell density near the visual streak (equatorial retina ~1 mm below the ONH), with a more diffuse distribution in the periphery (Figure 5E). In macaques, TH^+^cells were most concentrated in a ring with 2–3 mm radius surrounding the fovea (Figure 5F), consistent with previous reports [39]. However, their density declined markedly toward the mid-peripheral regions of the retina (Figure 5G). Note that the color scales in k-neighbor maps are not directly comparable across species due to radius adjustments for optimal visualization (mouse: 375 μm; rat and TLGS: 700 μm; macaque: 415 μm).

The distribution of dTH^+^cells also varied markedly among species. Mouse retinas lacked dTH^+^cells (Figure 5C’), while rat retinas exhibited almost exclusive dorsal localization (Figure 5D’). In TLGS retinas, dTH^+^cells were concentrated in more central regions but lacked a distinct correlation with the visual streak (Figure 5E’). In macaque retinas, dTH^+^cells were denser toward the macula but did not form the clear ring observed in their INL counterpart (Figure 5F’), and their density did not change markedly toward the periphery (Figure 5G’).

Analysis of parallel RGC distribution revealed that in rodents, RGCs clustered in the equatorial retina, peaking above the ONH in mouse and rat, and toward the temporal retina (Figure 5C″,D″) consistent with previous reports [40,41,42]. In TLGS, RGCs peaked below the ONH (Figure 5E″), in line with their visual streak [38,43]. In macaques, RGCs were concentrated in the macula, absent from the fovea, and decreased gradually toward the periphery (Figure 5F″) [38,44].

## 3. Discussion

Although DACs have been identified across multiple mammalian species [45,46,47,48,49,50,51,52,53,54,55,56], comprehensive comparative analyses of their total numbers, laminar localization, and topographical distribution have remained limited. Our study provides a detailed quantification and spatial characterization of TH^+^cells in four mammalian models—mouse, rat, squirrel, and macaque. This analysis highlights both conserved features and species-specific differences, particularly between nocturnal and diurnal mammals.

### 3.1. Validation of the TH Antibody for Labeling DACs in Different Species

The specificity of the TH antibody for DACs is supported by its co-localization with phenotypically identified (eGFP-positive large-soma cells) and morphologically con-firmed (wide-field structure revealed by neurobiotin injection and streptavidin labeling) DACs in the *Drd2*-eGFP mouse line (Figure 1) [37]. Although previous studies using conditional TH knockout models have reported faint TH immunoreactivity in tissues such as the adrenal gland, suggesting low-level expression in other cell types [57], retinal TH labeling is consistently restricted to a morphologically distinct population of amacrine cells. This supports the use of TH immunostaining as a reliable marker for DACs.

However, it is important to acknowledge that TH immunolabeling may not be exclusive to this cell type. Type II catecholaminergic neurons, non-dopaminergic cells that transiently express TH, have been described and low-level staining may be observed. In our own observations, faint TH immunoreactivity was occasionally detected in rat and macaque retinas. Despite this, DACs present a distinctive morphology and soma size that is consistent across species, enabling confident identification. Thus, while minor off-target labeling cannot be entirely excluded, the strong correspondence between TH immunoreactivity and the characteristic features of DACs in our study supports the continued use of TH as a marker for this cell type, with appropriate caution when interpreting absolute cell numbers, particularly in species where independent validation is limited.

### 3.2. Comparative Quantification of TH^+^Dopaminergic Amacrine Cells Across Species

The total number and density of TH^+^cells across different mammalian species revealed notable differences in their density and overall abundance. Among the species examined, mice had the fewest TH^+^cells overall (∼615, consistent with previous reports [13,58]) likely due to their relatively small retinal area; however, they exhibited the highest average density per mm^2^ in the INL. As expected, total number of TH^+^cells found in each species increased with retinal area. Rats showed a modest increase in total TH^+^cells (~900), while TLGSs showed a threefold increase in total TH^+^cells (~2400). Macaque retinas, though only partially quantified, exhibited high local densities, particularly around the macular region, consistent with previous descriptions [39] (Table 2). The overall density of DACs was similar between rats and TLGSs, while mouse and macaque retinas exhibited higher density as previously reported [26,39,59]. Interestingly, the proportion of dTH^+^cells, located in the GCL varied dramatically among species: virtually absent in mice, scarce in rats (~1.6%), but significantly more frequent in TLGSs (~6%) and in macaques (12%). This finding expands on prior descriptions of DAC stratification in these species [60,61] and supports the hypothesis that dopaminergic modulation might play an alternative functional role in diurnal species or those with higher visual acuity. Despite the existence of these two populations, both appear to arborize in the same sub-laminae of the INL suggesting similar functional role [49,50,52]. Thus, the higher density of TH^+^cells in macaques suggests an evolutionary increase in DAC populations in species with more complex visual processing demands; however, the elevated density of TH^+^cells in the INL of mouse retina may compensate for the lack of dTH^+^cells in the GCL.

### 3.3. Topographic Specializations TH^+^Dopaminergic Amacrine Cells

Across all species, TH^+^cells were primarily located in the INL and displayed widespread dendritic arbors within the outer sublamina of the inner plexiform layer (IPL, Figure 2), covering the retina without major gaps. While the general distribution was relatively uniform, higher densities were observed toward the central retina, consistent with previous descriptions in mouse [62,63], TLGS [53], and primate retinas [39,54,55,64]. However, species-specific topographical variations were evident. In TLGS and macaque retinas TH^+^cells density is consistent with the region’s enhanced visual function. In macaque retinas, TH^+^cell density peaked in a ring surrounding the foveal center (~2–3 mm radius in the macular region), as previously noted [39,54,55,64] (Figure 5F). In TLGS retinas, the highest density aligned with the visual streak (Figure 5E), an area associated with higher visual acuity due to increased cone and RGC densities [38,43]. Interestingly, this distribution resembled the pattern of DACs in rabbits [55], who also exhibit a visual streak. This strategic positioning suggests a role in enhancing dopamine modulation in regions of increased visual throughput. Mice, showed a slightly higher concentration in the mid-ventral retina (Figure 5C) coinciding with the peak density of cone photoreceptors [65]. In contrast, rats exhibited elevated TH^+^cell densities in mid-dorsal regions (Figure 5D) as previously reported [55]. This dorsal concentration of TH^+^cells does not align with the regions of peak RGC or cone photoreceptor density [66]. Instead, it may correspond to areas with higher densities of melanopsin-expressing intrinsically photosensitive RGCs (ipRGCs) in rats [67,68]. A similar topographic relationship is observed in the macaque retina, where melanopsin^+^ipRGCs are most densely concentrated in the central retina [69], potentially coinciding with the peak in DAC density. In contrast, this localization is not evident in the mouse retina, where ipRGCs show a modest mid-periphery and dorsal bias [70,71]. For the TLGS, such comparisons remain limited, as currently available antibodies do not effectively label the ipRGC population in this species.

The presence and localization of dTH^+^cells in the GCL also varied strikingly. In both squirrels and macaques, dTH^+^cells were centrally concentrated. In macques, they were distributed around the macula, though they did not form the same ring-like structure observed in their orthotopic INL counterparts (Figure 5F’). In squirrels, centrally located dTH^+^cells were present but did not align with the visual streak (Figure 5E’). In mice, dTH^+^cells were absent [60] (Figure 5C’), likely because the high density of TH^+^cells in the INL sufficiently supports dopaminergic modulation, obviating the need for a displaced subpopulation found in other species. It is plausible that in mice, dopaminergic influence on retinal circuitry is effectively mediated solely by INL DACs, reducing evolutionary or developmental pressure for generating dDACs. In rats, the existence of dTH^+^cells has been reported [61], and our results show that their extreme dorsal localization exceeds that of their counterpart in the INL (Figure 5D’). This unusual pattern mirrors the distribution seen in guinea pig retinas [55]. Despite these species-specific variations, dDACs have been reported to ramify in the same IPL strata as their orthotopic counterparts [49,50,52], reinforcing the notion of a conserved role in global retinal modulation rather than strict regional specialization. Nonetheless, the species-dependent regionalization—central enrichment in TLGS and macaque, ventral preference in mouse, and dorsal concentration in rat—may reflect nuanced, region-specific functional roles in modulating retinal output. This possibility warrants further investigation, particularly in terms of synaptic connectivity and the spatial relationship between TH^+^ dendritic fields and RGC populations.

In perspective, the relatively low and stable density of DACs becomes more apparent when contrasted with the more dramatic variations in Brn3a^+^RGC densities across species. In mice and rats, RGCs densities peaked above the ONH [40,41,42], whereas in TLGSs the highest RGC density was located below the ONH along the visual streak as previously reported [38,72]. In macaques, RGCs were concentrated in the macula but sharply declined in the foveal center, consistent with the well-established primate retinal topography [44,72]. These findings underscore the spatial divergence between the more uniform distribution of TH^+^cells and the highly regionalized distribution of RGCs, which likely reflects distinct functional specializations.

In all species, the widespread dendritic arbors of TH^+^cells extend from the peripheral retinal rim to the peripapillary region surrounding—but not penetrating—the ONH itself (Figure 4). This extensive coverage suggests a role in modulating retinal activity over broad areas. Given that the ONH is the convergence point for all RGC axons exiting the eye, the proximity of TH^+^ dendrites to this region may facilitate RGC synchronization and contribute to coordinated visual processing. Although, TH^+^cell processes do not contact the central retinal artery directly, their close proximity raises the possibility that dopaminergic signaling could influence local vascular dynamics, potentially affecting retinal metabolism and function. While direct evidence for the DAC-mediated modulation of retinal blood flow is limited, studies have demonstrated that the stimulation of certain amacrine cell types can impact capillary perfusion [73]. These observations underscore the need for further research to elucidate the multifaceted roles of DACs in retinal physiology.

### 3.4. Ratio of TH^+^Dopaminergic Amacrine Cells to Retinal Ganglion Cells

It is notable that although the absolute number of TH^+^cells increases from mouse to rat, and further to TLGS and macaque, this rise is substantially outpaced by the exponential increase in RGCs—ranging from ~46,500 in mouse, ~84,000 in rat, ~607,000 in TLGS, to over 1–1.2 million in primates [44,72]. As a result, the relative representation of dopaminergic modulation diminishes, particularly in diurnal species. While the overall ratio of TH^+^cells to Brn3a^+^RGCs remains relatively consistent (~1:80) in mouse, rat and macaque (based on partial retina quantifications), TLGS displays a markedly lower ratio of approximately 1:253 (Table 2). This reduced ratio in TLGS may reflect species-specific adaptations in retinal circuitry, likely related to their diurnal, cone-rich visual system [36]. Unlike nocturnal rodents, TLGSs rely heavily on cone-mediated vision and possess a pronounced visual streak [38,43], potentially reducing the need for dense dopaminergic modulation that primarily supports light adaptation and circadian regulation in rod-dominant retinas. Alternatively, the lower TH^+^cell density relative to RGCs may be functionally offset by enhanced dopaminergic release per cell, greater synaptic coverage, or increased receptor expression in post-synaptic targets, thereby maintaining effective neuromodulatory influence despite fewer DACs. Another possibility is that, due to the low proportion of rods in TLGS retinas [43], dopamine’s modulatory role—typically involved in adjusting rod input to cone pathways—may be less critical, resulting in a more specialized role in regulating cone-cone gap junctions in this cone-dominant species [74]. These specific roles may not require the same cellular density observed in nocturnal species, where precise dopaminergic control over rod pathway adaptation is critical for scotopic vision. Additionally, a broader diffusion range of dopamine or tighter circadian control of dopamine release could allow fewer DACs to cover larger retinal areas efficiently.

Taken together, these findings suggest that the TH^+^cell/RGC ratio may not scale uniformly across species but rather adapt to the ecological and visual demands of the organism. The significantly lower ratio in TLGS may thus represent an evolutionary specialization that balances neuromodulatory efficiency with structural economy in cone-dominated retinas. Future studies investigating dopaminergic receptor distribution and dopamine turnover in diurnal versus nocturnal species could further elucidate these functional differences.

While our study quantifies TH^+^cells relative to Brn3a^+^RGCs, we acknowledge that DACs are functionally more closely associated with AII amacrine cells, particularly in the modulation of rod–cone circuitry. However, due to the lack of a specific and validated marker for AII amacrine cells across all species examined, we were unable to include these cells as a reference population. We selected Brn3a^+^RGCs as a consistent and well-characterized anatomical baseline for cross-species comparisons. Future studies may benefit from incorporating additional amacrine subtypes when suitable markers become available, to further refine our understanding of DAC distribution and function.

### 3.5. Functional Implications and Considerations in Evolutionary Visual Processing and Dysfunction

In this comparative study, we identified both conserved and species-specific anatomical features of TH^+^cells across four mammalian species, offering new insights into the evolutionary role of retinal dopamine. Our findings show that TH^+^cells are consistently localized to the inner nuclear layer (INL), with a broad dendritic arbor extending along the retinal surface in the outer sublamina of the IPL, supporting their role in the wide-field modulation of retinal circuitry. This architecture suggests conserved functions in contrast sensitivity, circadian regulation, and light adaptation [1,2,3,4,5]. However, beyond differences attributable to retinal size, species varied markedly in TH^+^cell density, topographic distribution, and presence of dTH^+^cells in the GCL. These variations point to regional enrichment of TH^+^cells in retinal zones critical for visual processing and may reflect evolutionary adaptations to distinct visual demands, particularly in species with greater visual complexity.

Moreover, species-specific differences in laminar distribution and TH^+^cell/RGC ratios likely reflect adaptations to diverse visual processing demands. In small nocturnal mammals such as mice, a higher TH^+^cell-to-RGC ratio may enhance modulatory control to compensate for lower overall visual acuity. In contrast, species with more complex visual systems—such as TLGS and macaques (particularly in the macular region)—maintain relatively fewer TH^+^cells per RGC. This may reflect a shift toward more specialized and diverse interneuronal networks for retinal processing. Future studies examining the functional consequences of these anatomical differences could provide deeper insights into the role of dopamine in species-specific visual processing strategies.

These findings suggest that while the fundamental architecture of the dopaminergic system in the retina is conserved, its species-specific specialization—particularly in topographic distribution—reflects evolutionary and functional adaptations to the unique visual ecology and demands of each species. These differences emphasize the importance of considering species-specific retinal architectures when extrapolating experimental findings and in the design of interventions targeting retinal neuromodulation. By revealing how dopamine-mediated modulation scales and localizes differently across mammals, this comparative framework deepens our understanding of retinal circuitry and sets the stage for exploring species-dependent vulnerabilities to retinal diseases involving dopaminergic dysfunction.

The high density of TH+cells in the central retina of macaques, particularly around the macula, may have important implications for understanding visual dysfunction, such as PD and diabetic retinopathy. As dopamine plays a critical role in regulating contrast sensitivity, light adaptation, and color discrimination [1,2,3,4,5], the loss or dysfunction of DACs may contribute to the visual symptoms commonly reported in PD patients [75,76,77,78,79,80,81,82,83,84,85]. The macula is central to high-acuity vision, and our finding that macaques exhibit a pronounced central enrichment of TH+cells raises the possibility that this region may be especially susceptible to dopaminergic deficits in disease states. These observations suggest that species-specific differences in DAC distribution, such as the lower central density observed in rodents, could partly explain why visual deficits in PD are more difficult to model in non-primate species. However, it is important to note that while the macaque retina closely resembles that of humans, subtle interspecies differences in retinal architecture, cell density, or dopaminergic signaling cannot be excluded. Therefore, further studies using human postmortem tissue will be critical to confirm whether the patterns observed in macaques are conserved in humans and relevant to the pathophysiology of retinal involvement in PD.

## 4. Materials and Methods

### 4.1. Animal Handling

Each specie and strain were cared for following specific protocols (NEI ASP#606, #595, and NINDS ASP#1344 for mouse, TLGS, and the *Drd2*-eGFP mouse line, respectively). All procedures were conducted according to protocols approved by the National Eye Institute Animal Care and Use Committee and adhered to the Association for Research in Vision and Ophthalmology (ARVO) Statement for the Use of Animals in Ophthalmic and Vision Research. Additionally, this study was conducted in accordance with U.S. laws and regulations set forth by the U.S. Department of Agriculture.

Adult pigmented mice (C57BL/6J, 3-month-old female and male, n = 11 eyes) were obtained from the National Eye Institute (NEI) breeding colony. Adult *Drd2-*eGFP transgenic mice (2-month-old female and male, n = 6 eyes), which express enhanced green fluorescent protein (EGFP) under the control of the dopamine receptor D2 (*Drd2*) promoter, were obtained from the National Institute of Neurological Disease and Stroke (NINDS). Adult thirteen-lined ground squirrels (hence TLGS, ~2-year-old female and male, n = 6 eyes) were obtained from a breeding colony at the University of Wisconsin Oshkosh. Postmortem eyes from adult albino rats (Sprague Dawley, 9-month-old female, n = 10 eyes) were obtained from Megan Kopera from the National Eye Institute (NEI). Postmortem eyes from macaque (Rhesus, 8–12-year-old female and male, n = 4 eyes) were obtained from the Diagnostic and Research Services Branch of the National Institutes of Health (NIH).

Mice and TLGS were housed in a controlled environment with regulated temperature and lighting, maintaining a 12 h light/dark cycle at 22 °C, with unrestricted access to food and water. During winter, TLGSs were transferred to a hibernaculum set at 4 °C, as per established guidelines [86].

### 4.2. Whole-Cell Patch Clamp and Dye Loading for TH Antibody Characterization

*Drd2*-eGFP mice [37] were used to evaluate the specificity of the TH antibody in identifying DACs. The retina of this mouse identifies two distinct cell populations that express eGFP, but the DACs can be phenotypically identified because of their larger soma. Mice were euthanized and retinas dissected freshly with four relieving cuts and placed flat onto a poly-D-lysine-coated glass coverslip (12 mm diameter, Corning BioCoat Cellware) that was secured to a recording dish via grease (Dow Corning); a harp (ALA Scientific, HSG 5A) was put over the tissue. Retinas were mounted photoreceptor side down. Tissues were perfused with Ames medium (285 mOsm, 7–9 mL/min) maintained at a temperature of 30–32 °C. Large GFP positive somas (20–25 µm diameter) located in the INL were targeted using multi-photon laser imaging (920 nm) for morphological characterization. Target cells were patched with 6–8 MΩ glass pipettes containing (in mM): 123 KCH 3SO 3, 10 HEPES, 1 MgCl 2, 1 NaCl, 2 EGTA, 7 Phosphocreatine di (tris), 4 Mg-ATP, 0.5 Na-GTP, and 3% neurobiotin (Vector Labs SP-1150) and subsequently fixed for immunodetection with streptavidin. For this characterization, 8 cells were injected from 6 retinas.

### 4.3. Tissue Fixation and Preparation

C57BL/6J mice and TLGSs and were euthanized in the morning by CO_2_, followed by trans-cardiac perfusion with a fixative solution consisting of 0.9% saline solution and 4% paraformaldehyde in 0.1 M phosphate buffer. Rat and monkey eyes were collected postmortem and submerged in the same fixative solution for 1 h. To preserve retinal orientation, before enucleating the eyes, a burn point was placed in the dorsal pole. Later, the retinas were carefully isolated from the eyecup and flattened as whole mounts by making radial cuts. Another set of retinas (n = 3 eyes/specie) were cryoprotected in a graded sucrose series (15% and 30% sucrose in PBS) and embedded in optimal cutting temperature (Sakura Finetek, Torrance, CA, USA) at −80 °C for cryo-sectioning (Leica, CM3050S) at 16 μm thickness following the dorso-ventral (DV) axis.

### 4.4. Immunostaining

Retinas were permeabilized in PBS containing 0.5% Triton X-100 (PBS-T) four times for 10 min each at RT. To prevent non-specific binding, primary antibodies were incubated in a blocking solution supplemented with 2% normal donkey serum overnight at RT with gentle agitation. For retinal sections and whole-mount retinas, Tyrosine hydroxylase, RBPMS or Brn3a, and Choline Acetyltransferase primary antibodies were used to label general populations of DACs, RGCs and Starburst Amacrine cells (Mouse α-TH, Millipore MAB5280, 1:500, [87]; Rabbit α-RBPMS, GeneTex GTX118619, 1:500, [88]; Goat α-Brn3a, SantaCruz Biotechnologies (C-20) sc-31984, 1:500, [40,88]; goat α-ChAT, Millipore AB144P, 1:250, [38]), respectively. Additionally, for *Drd2*-eGFP retinas injected with neurobiotin were immunodetected with Cy3-conjugated Streptavidin (Jackson ImmunoResearch 016–160-084, 1:250) and a TH antibody (Sheep α-TH, Millipore Sigma AB1542, [89]), which produced staining consistent with that of the mouse anti-TH antibody. Then, the samples were washed four times in PBS-T for 10 min each followed by incubation with the appropriate fluorophore-conjugated secondary antibodies (Donkey α-mouse, α-goat or α-rabbit, Jackson Immunoresearch, 1:500 diluted in PBS-T) overnight at RT in the dark. Retinas were flattened and mounted on glass slides with the ganglion cell layer facing upward. Samples were coverslipped using an antifade mounting medium and stored at −20 °C until imaging. Retinal sections were additionally counterstained with DAPI to visualize nuclei. Negative controls were performed by omitting the primary antibody, confirming the specificity of the immunolabeling. However, nonspecific binding was occasionally observed in the retinal vasculature of rat and macaque samples, likely due to the absence of cardiac perfusion prior to fixation.

RBPMS and Brn3a were both used to label retinal ganglion cells (RGCs) due to their complementary advantages. RBPMS stains the cytoplasm of all RGCs, making it useful for confirming that a TH^+^cell is not an RGC and for visualizing RGC localization and morphology in retinal sections. However, its diffuse cytoplasmic signal makes automated quantification difficult in regions with a high cell density [88]. Brn3a, a nuclear transcription factor expressed in the majority of RGCs (92–96% depending on species [88]), provides sharp nuclear labeling that facilitates accurate automated quantification [88]. For this reason, Brn3a was used in analyses requiring precise cell counts only, while RBPMS served as a morphological and spatial reference.

### 4.5. Image Acquisition and Processing

Immunofluorescence was visualized using a confocal microscope (Zeiss LSM 780). A 20× objective was used to acquire z-stacks covering the entire retina in a zig-zag pattern, ensuring complete coverage for photomontage reconstruction. This approach was applied to whole-mounted retinas from mouse, rat, and TLGS. For macaque samples, where full retinas were not available, three large retinal pieces (each from a different eye) were imaged using the same protocol. Two of these samples contained mid-peripheral and peripheral regions, while the third included the macula, fovea, and adjacent areas.

Retinal sections were taken with a 40× objective to capture detailed views of DACs, RGCs and Starburst Amacrine cells. High magnification images (40×, 0.18 mm^2^) from flattened retinas were acquired in three different retinal regions for each species. In mouse and rat retinas, images were taken from the dorsal retina (0.5–1 mm from the peripheral edge), the central retina (0.5–1 mm above the optic nerve head [ONH]), and the mid-ventral retina (1.5–2.5 mm below the ONH), respectively. In 13-lined ground squirrel (TLGS) retinas, images were obtained from the dorsal retina (2 mm from the periphery), the visual streak (1 mm below the ONH), and the mid-ventral retina (4 mm below the ONH). In macaque retinas, images were acquired from the peripheral retina (2 mm from the edge), the macula, and the mid-retina (4 mm from the center of the macula). TH^+^ and Brn3a^+^RGC densities were based on one representative image taken from each of the three defined retinal regions per retina (n = 4 for mouse, n = 4 for rat, n = 3 for TLGS, and n = 4 for macaque).

To further enhance the visibility of dendritic arborization, contrast-enhanced grayscale images of TH staining were prepared and overlaid with DAPI counterstaining (pseudo-colored in yellow) to delineate retinal layers. These adjustments were made using Adobe Photoshop, applying uniform contrast enhancement and color assignment across images for consistency.

### 4.6. Quantification and Spatial Distribution Analysis

To estimate cell density across retinal regions (dorsal/periphery, central/visual streak/macula and mid-ventral-middle), TH^+^cells were manually identified and quantified, while Brn3a^+^RGCs were automatically quantified except those corresponding to the macula of the NHP that were manually dotted to maximize accuracy. For total counts and topographical analysis, TH^+^DACs were manually marked in the high-resolution retinal photomontages using image editing software (Adobe Photoshop v26.6.1). Multiple z-planes were examined to distinguish between TH^+^DACs located in the INL from those in the GCL. These manually annotated cells (dots representing TH^+^cells in INL or GCL), along with Brn3a^+^RGCs, were subsequently quantified using an automated algorithm in FIJI (NIH) previously validated (Pearson correlation R^2^ = 0.97) [38]. Briefly, after background subtraction (rolling ball radius: 50 pixels), images were converted to 8-bit grayscale and thresholded to isolate signal from noise. Binary images were processed with watershed segmentation and a median filter to separate touching nuclei and remove small artifacts. Only particles within a defined size and shape range were counted as positive cells. Spatial coordinates and total cell counts were extracted and exported to a spreadsheet for further spatial analysis [67,90]. The number of nearest neighbors for each TH^+^DACs was calculated within a fixed radius—525 µm for mouse; 700 µm for rat and TLGS; and 415 µm for macaque retinas. To visualize spatial distribution, k-nearest neighbor plots and isodensity maps were generated using Sigma Plot 11, providing detailed insight into the topographical organization of TH^+^DACs and Brn3a^+^RGCs across species [38].

### 4.7. Statistical Analysis

Statistical analyses were performed using GraphPad Prism 10.4.0. Comparisons among groups were conducted using one-way ANOVA, and differences were considered statistically significant for *p*-values < 0.05. Data are presented as mean ± standard deviation (SD).

## Figures and Tables

**Table 1:** Density and ratio of TH^+^DACs to Brn3a^+^RGCs along the dorso-ventral axis across mammalian species. The average densities (cell/mm^2^) of TH^+^cells and Brn3a^+^RGCs were analyzed using images of identical frame size (0.18 mm^2^), acquired by focusing on different retinal layers—(INL for DACs and GCL for RGCs).

		C57 Mouse	SD Rat	TLGS	Macaque NHP
Dorsal/Periphery	TH^+^cells	40 ± 9	22 ± 8	22 ± 6	51 ± 5
	Brn3a^+^RGCs	2170 ± 299	1192 ± 280	1782 ± 202	136 ± 126
Center/Macula	TH^+^cells	55 ± 8	26 ± 5	31 ± 6	75 ± 11
	Brn3a^+^RGCs	5981 ± 557	3060 ± 162	15,738 ± 1266	15,538 ± 4701
Mid-ventral	TH^+^cells	44 ± 10	26 ± 8	30 ± 3	32 ± 13
	Brn3a^+^RGCs	4693 ± 732	1839 ± 120	4529 ± 835	1971 ± 795
Average	TH^+^cells	47 ± 11	25 ± 7	28 ± 6	53 ± 21
	Brn3a^+^RGCs	4281 ± 1729	2030 ± 829	7350 ± 6448	5882 ± 7595
Ratio	TH^+^cell/RGC	1:91	1:81	1:265	1:112

**Table 2:** Total number, retinal area and average density of TH^+^cells and Brn3a^+^RGCs in mouse, rat, ground squirrel and macaque retinas. Data are presented as mean ± SD. Sample sizes: mouse, n = 8 retinas; rat, n = 7 retinas; TLGS, n = 3 retinas; macaque, n = 3 retinal pieces, each obtained from a different eye. * *Note*: In macaque (non-human primates, NHPs), the total number and density of TH^+^DACs and Brn3a^+^RGCs were quantified from three large retinal pieces with an average area of 46 ± 28 mm^2^. Two of these samples included retina spanning from the center to the periphery, while the third encompassed the central retina (including macula and fovea). Unlike in mouse, rat, and squirrel, macaque measurements were not taken from the entire retina. Although it would be feasible to estimate total cell numbers in the macaque retina from the analyzed samples, the strong regional specialization, particularly in and around the macula, could result in significant over- or underestimation of the actual number of TH^+^cells or RGCs. To prevent the introduction of potentially misleading values, we have chosen to report accurate average densities instead.

			C57 Mouse	SD Rat	TLGS	Macaque NHP *
Total number	TH^+^cells	INL	616 ± 33	993 ± 123	2400 ± 262	1298 ± 1405 *
		GCL	0 ± 0 (0%)	16 ± 3 (1.6%)	154 ± 8 (6%)	79 ± 26 * (12.4%)
	Brn3a^+^RGCs	GCL	46,497 ± 1768	84,478 ± 3268	607,661 ± 17,557	87,585 ± 121,811 *
Retinal area (mm^2^)			16 ± 1	59 ± 4	177 ± 9	46 ± 28 *
Average density (cell/mm^2^)	TH^+^cells	INL	38 ± 1	17 ± 2	14 ± 2	29 ± 18 *
		GCL	0 ± 0	0.3 ± 0	0.8 ± 0	2 ± 1*
	Brn3a^+^RGCs	GCL	3084 ± 117	1652 ± 106	3754 ± 171	1860 ± 2209 *
Ratio	TH^+^cell/RGC		1:76	1:85	1:253	*1:79*

## Data Availability

The data supporting the findings of this study are available from the corresponding authors upon reasonable request.

## Abbreviations

The following abbreviations are used in this manuscript:

Brn3a	Or POU4F1, a class IV POU domain-containing transcription factor
DACs	Dopaminergic amacrine cells
dDACs	Displaced Dopaminergic amacrine cells
GCL	Ganglion Cell Layer
INL	Inner Nuclear Layer
IPL	Inner Plexiform Layer
ipRGCs	Intrinsically photosensitive RGCs
NHP	Non-human primate
RBPMS	RNA-binding protein with multiple splicing
RGCs	Retinal ganglion cells
TH	Tyrosine hydroxylase
TLGS	Thirteen-Lined Ground Squirrel
